# Ethyl 1-(4-meth­oxy­benz­yl)-3-*p*-tolyl-1*H*-pyrazole-5-carboxyl­ate

**DOI:** 10.1107/S1600536811012311

**Published:** 2011-04-13

**Authors:** Chuan-Xing Shi, Yun-Man Xie

**Affiliations:** a Henan Chemical Industry Research Institute Co. Ltd, Zheng Zhou 450052, People’s Republic of China

## Abstract

In the title compound, C_21_H_22_N_2_O_3_, the pyrazole ring makes dihedral angles of 12.93 (8) and 69.38 (8)°, respectively, with the tolyl and meth­oxy­benzyl rings.

## Related literature

For the pharmacological activity of pyrazole compounds, see: Ge *et al.* (2009 ▶, 2011 ▶). For the synthesis of the title compound, see: Li *et al.* (2011 ▶). For the structure of ethyl 1-benzyl-3-(4-fluoro­phen­yl)-1*H*-pyrazole-5-carboxyl­ate, see: Han *et al.* (2011 ▶). For a related structure, see: Ge *et al.* (2007 ▶). 
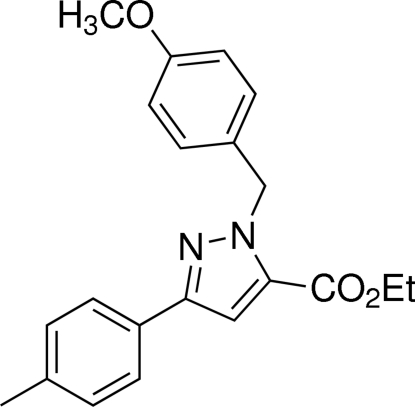


## Experimental

### 

#### Crystal data

C_21_H_22_N_2_O_3_
*M* *_r_* = 350.41
Monoclinic, 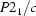

*a* = 7.3272 (6) Å
*b* = 24.8129 (19) Å
*c* = 10.4556 (8) Å
β = 97.391 (1)°
*V* = 1885.1 (3) Å^3^
*Z* = 4
Mo *K*α radiation
μ = 0.08 mm^−1^
*T* = 298 K
0.23 × 0.16 × 0.13 mm

#### Data collection

Bruker SMART CCD area-detector diffractometer
Absorption correction: multi-scan (*SADABS*; Bruker, 1998 ▶) *T*_min_ = 0.981, *T*_max_ = 0.989
9809 measured reflections
3352 independent reflections
2678 reflections with *I* > 2σ(*I*)
*R*_int_ = 0.022

#### Refinement

*R*[*F* ^2^ > 2σ(*F* ^2^)] = 0.042
*wR*(*F* ^2^) = 0.119
*S* = 1.03
3352 reflections
235 parameters
H-atom parameters constrained
Δρ_max_ = 0.19 e Å^−3^
Δρ_min_ = −0.18 e Å^−3^

### 

Data collection: *SMART* (Bruker, 1998 ▶); cell refinement: *SAINT* (Bruker, 1998 ▶); data reduction: *SAINT*; program(s) used to solve structure: *SHELXS97* (Sheldrick, 2008 ▶); program(s) used to refine structure: *SHELXL97* (Sheldrick, 2008 ▶); molecular graphics: *XP* in *SHELXTL* (Sheldrick, 2008 ▶); software used to prepare material for publication: *SHELXL97*.

## Supplementary Material

Crystal structure: contains datablocks I, global. DOI: 10.1107/S1600536811012311/jh2273sup1.cif

Structure factors: contains datablocks I. DOI: 10.1107/S1600536811012311/jh2273Isup2.hkl

Additional supplementary materials:  crystallographic information; 3D view; checkCIF report

## Figures and Tables

**Fig. 1.**
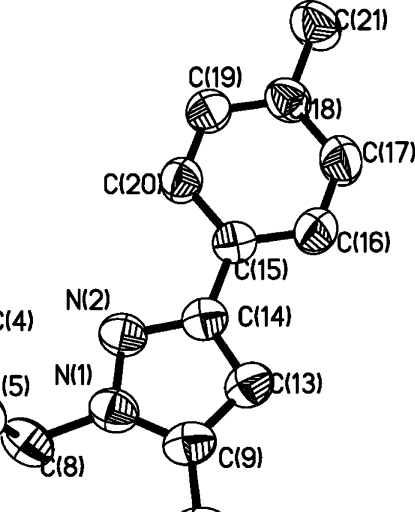
The molecular structure of (I), showing displacement ellipsoids drawn at the 50% probability level.

## supplementary crystallographic information

### Comment

Synthesis of nitrogen-containing heterocyclic compounds has been a subject of great interest due to the wide application in agrochemical and pharmaceutical fields (Ge *et al.*; 2011, 2009). Some pyrazole derivatives which belong to this category have been of interest for their biological activities. Considerable efforts have been devoted to the development of novel pyrazole compounds. We report here the crystal structure of the title compound, (I) (Fig. 1)

### Experimental

A mixture of ethyl 3-*p*-tolyl-1*H*-pyrazole-5-carboxylate (0.02 mol), 1-(chloromethyl)-4-methoxybenzene (0.0024 mol) and potassium carbonate (0.02 mol) in acetonitrile (100 ml) was heated to reflux for 10 h. The solvent was removed under reduced pressure and an product was isolated by column chromatography on silica gel (yield 85%). Crystals of (I) suitable for X-ray diffraction were obtained by slow cooling of the refluxed solution of the product in ethyl acetate at room temperature for 2 d.

### Refinement

All H atoms were placed in geometrically calculated positions and refined using a riding model with C—H = 0.97 Å (for CH_2_ groups) and 0.96 Å (for CH_3_ groups), their isotropic displacement parameters were set to 1.2 times (1.5 times for CH_3_ groups) the equivalent displacement parameter of their parent atoms.

### Crystal data

**Table:**

C_21_H_22_N_2_O_3_	*F*(000) = 744
*M**_r_* = 350.41	*D*_x_ = 1.235 Mg m^−^^3^
Monoclinic, *P*2_1_/*c*	Mo *K*α radiation, λ = 0.71073 Å
Hall symbol: -P cd ..2ybc	Cell parameters from 4354 reflections
*a* = 7.3272 (6) Å	θ = 2.6–27.0°
*b* = 24.8129 (19) Å	µ = 0.08 mm^−^^1^
*c* = 10.4556 (8) Å	*T* = 298 K
β = 97.391 (1)°	Block, colorless
*V* = 1885.1 (3) Å^3^	0.23 × 0.16 × 0.13 mm
*Z* = 4	

### Data collection

**Table:**

Bruker SMART CCD area-detector diffractometer	3352 independent reflections
Radiation source: fine-focus sealed tube	2678 reflections with *I* > 2σ(*I*)
graphite	*R*_int_ = 0.022
φ and ω scans	θ_max_ = 25.1°, θ_min_ = 2.1°
Absorption correction: multi-scan (*SADABS*; Bruker, 1998)	*h* = −8→8
*T*_min_ = 0.981, *T*_max_ = 0.989	*k* = −27→29
9809 measured reflections	*l* = −12→12

### Refinement

**Table:**

Refinement on *F*^2^	Primary atom site location: structure-invariant direct methods
Least-squares matrix: full	Secondary atom site location: difference Fourier map
*R*[*F*^2^ > 2σ(*F*^2^)] = 0.042	Hydrogen site location: inferred from neighbouring sites
*wR*(*F*^2^) = 0.119	H-atom parameters constrained
*S* = 1.03	*w* = 1/[σ^2^(*F*_o_^2^) + (0.0599*P*)^2^ + 0.3339*P*] where *P* = (*F*_o_^2^ + 2*F*_c_^2^)/3
3352 reflections	(Δ/σ)_max_ = 0.001
235 parameters	Δρ_max_ = 0.19 e Å^−^^3^
0 restraints	Δρ_min_ = −0.18 e Å^−^^3^

### Special details

**Table:**

Geometry. All e.s.d.'s (except the e.s.d. in the dihedral angle between two l.s. planes) are estimated using the full covariance matrix. The cell e.s.d.'s are taken into account individually in the estimation of e.s.d.'s in distances, angles and torsion angles; correlations between e.s.d.'s in cell parameters are only used when they are defined by crystal symmetry. An approximate (isotropic) treatment of cell e.s.d.'s is used for estimating e.s.d.'s involving l.s. planes.
Refinement. Refinement of *F*^2^ against ALL reflections. The weighted *R*-factor *wR* and goodness of fit *S* are based on *F*^2^, conventional *R*-factors *R* are based on *F*, with *F* set to zero for negative *F*^2^. The threshold expression of *F*^2^ > σ(*F*^2^) is used only for calculating *R*-factors(gt) *etc*. and is not relevant to the choice of reflections for refinement. *R*-factors based on *F*^2^ are statistically about twice as large as those based on *F*, and *R*- factors based on ALL data will be even larger.

### Fractional atomic coordinates and isotropic or equivalent isotropic displacement parameters (Å^2^)

**Table:**

	*x*	*y*	*z*	*U*_iso_*/*U*_eq_	
O1	0.88269 (19)	0.71616 (5)	1.05256 (13)	0.0819 (4)	
O2	1.43164 (19)	0.64241 (7)	0.60151 (14)	0.0970 (5)	
O3	1.36112 (17)	0.67333 (5)	0.40045 (14)	0.0775 (4)	
N1	1.08187 (17)	0.58456 (5)	0.56329 (12)	0.0554 (3)	
N2	0.91743 (17)	0.56391 (5)	0.51579 (12)	0.0538 (3)	
C1	0.9991 (4)	0.75363 (9)	1.1249 (2)	0.1030 (8)	
H1A	1.0928	0.7346	1.1799	0.155*	
H1B	0.9279	0.7751	1.1767	0.155*	
H1C	1.0555	0.7766	1.0674	0.155*	
C2	0.9582 (2)	0.68359 (6)	0.96740 (15)	0.0576 (4)	
C3	0.8353 (2)	0.65104 (7)	0.89121 (16)	0.0600 (4)	
H3	0.7105	0.6526	0.8994	0.072*	
C4	0.8966 (2)	0.61650 (6)	0.80360 (15)	0.0560 (4)	
H4	0.8125	0.5949	0.7528	0.067*	
C5	1.0821 (2)	0.61327 (6)	0.78944 (14)	0.0513 (4)	
C6	1.2021 (2)	0.64674 (7)	0.86452 (16)	0.0620 (4)	
H6	1.3265	0.6458	0.8551	0.074*	
C7	1.1422 (2)	0.68166 (7)	0.95353 (16)	0.0641 (4)	
H7	1.2258	0.7037	1.0036	0.077*	
C8	1.1502 (2)	0.57325 (7)	0.69837 (16)	0.0635 (4)	
H8A	1.2837	0.5738	0.7094	0.076*	
H8B	1.1114	0.5374	0.7200	0.076*	
C9	1.1513 (2)	0.61647 (6)	0.47571 (16)	0.0559 (4)	
C10	1.3294 (2)	0.64466 (8)	0.50244 (19)	0.0666 (5)	
C11	1.5375 (3)	0.70117 (9)	0.4111 (2)	0.0885 (7)	
H11A	1.6373	0.6762	0.4374	0.106*	
H11B	1.5415	0.7298	0.4745	0.106*	
C12	1.5551 (4)	0.72362 (11)	0.2822 (3)	0.1141 (9)	
H12A	1.5448	0.6951	0.2198	0.171*	
H12B	1.6728	0.7408	0.2840	0.171*	
H12C	1.4592	0.7495	0.2593	0.171*	
C13	1.0262 (2)	0.61579 (6)	0.36636 (16)	0.0565 (4)	
H13	1.0352	0.6336	0.2891	0.068*	
C14	0.8824 (2)	0.58284 (6)	0.39496 (14)	0.0493 (4)	
C15	0.7099 (2)	0.56794 (6)	0.31485 (14)	0.0485 (3)	
C16	0.6829 (2)	0.57760 (7)	0.18385 (16)	0.0659 (5)	
H16	0.7760	0.5936	0.1445	0.079*	
C17	0.5193 (3)	0.56376 (8)	0.11051 (17)	0.0725 (5)	
H17	0.5047	0.5704	0.0223	0.087*	
C18	0.3767 (2)	0.54022 (6)	0.16490 (16)	0.0592 (4)	
C19	0.4049 (2)	0.52993 (6)	0.29509 (16)	0.0585 (4)	
H19	0.3123	0.5134	0.3341	0.070*	
C20	0.5676 (2)	0.54355 (6)	0.36890 (15)	0.0569 (4)	
H20	0.5825	0.5363	0.4569	0.068*	
C21	0.1957 (3)	0.52677 (9)	0.08541 (19)	0.0802 (6)	
H21A	0.1329	0.4998	0.1291	0.120*	
H21B	0.2181	0.5133	0.0028	0.120*	
H21C	0.1211	0.5586	0.0738	0.120*	

### Atomic displacement parameters (Å^2^)

**Table:**

	*U*^11^	*U*^22^	*U*^33^	*U*^12^	*U*^13^	*U*^23^
O1	0.0878 (9)	0.0836 (9)	0.0793 (8)	−0.0158 (7)	0.0288 (7)	−0.0257 (7)
O2	0.0658 (8)	0.1384 (13)	0.0840 (10)	−0.0265 (8)	−0.0014 (7)	−0.0224 (9)
O3	0.0556 (7)	0.0793 (8)	0.0982 (10)	−0.0196 (6)	0.0123 (7)	−0.0081 (7)
N1	0.0511 (7)	0.0554 (7)	0.0582 (8)	0.0025 (6)	0.0013 (6)	−0.0091 (6)
N2	0.0500 (7)	0.0516 (7)	0.0588 (8)	−0.0014 (6)	0.0028 (6)	−0.0052 (6)
C1	0.128 (2)	0.0880 (15)	0.1008 (16)	−0.0380 (14)	0.0435 (14)	−0.0397 (13)
C2	0.0673 (10)	0.0547 (9)	0.0516 (9)	−0.0087 (8)	0.0112 (7)	0.0018 (7)
C3	0.0532 (9)	0.0632 (10)	0.0642 (10)	−0.0085 (8)	0.0104 (8)	0.0016 (8)
C4	0.0545 (9)	0.0524 (8)	0.0583 (9)	−0.0097 (7)	−0.0032 (7)	0.0014 (7)
C5	0.0542 (9)	0.0478 (8)	0.0490 (8)	0.0021 (7)	−0.0042 (6)	0.0061 (6)
C6	0.0476 (9)	0.0735 (11)	0.0622 (10)	−0.0030 (8)	−0.0030 (7)	−0.0022 (8)
C7	0.0627 (10)	0.0691 (10)	0.0584 (9)	−0.0155 (8)	−0.0007 (8)	−0.0095 (8)
C8	0.0607 (10)	0.0592 (9)	0.0665 (10)	0.0116 (8)	−0.0080 (8)	−0.0016 (8)
C9	0.0482 (8)	0.0564 (9)	0.0643 (10)	−0.0028 (7)	0.0113 (7)	−0.0149 (7)
C10	0.0503 (9)	0.0732 (11)	0.0767 (12)	−0.0049 (8)	0.0104 (9)	−0.0244 (10)
C11	0.0576 (11)	0.0855 (13)	0.1256 (19)	−0.0235 (10)	0.0243 (11)	−0.0262 (13)
C12	0.0986 (18)	0.1104 (18)	0.142 (2)	−0.0398 (15)	0.0482 (16)	−0.0131 (16)
C13	0.0533 (9)	0.0593 (9)	0.0582 (9)	−0.0068 (7)	0.0120 (7)	−0.0067 (7)
C14	0.0486 (8)	0.0466 (8)	0.0532 (8)	0.0003 (6)	0.0088 (6)	−0.0070 (6)
C15	0.0487 (8)	0.0434 (7)	0.0535 (8)	−0.0011 (6)	0.0072 (6)	−0.0031 (6)
C16	0.0593 (10)	0.0797 (11)	0.0590 (10)	−0.0138 (9)	0.0082 (8)	0.0101 (8)
C17	0.0699 (11)	0.0911 (13)	0.0535 (10)	−0.0144 (10)	−0.0031 (8)	0.0134 (9)
C18	0.0558 (9)	0.0548 (9)	0.0639 (10)	−0.0049 (7)	−0.0034 (8)	0.0031 (8)
C19	0.0534 (9)	0.0594 (9)	0.0623 (10)	−0.0107 (7)	0.0063 (7)	0.0046 (7)
C20	0.0584 (10)	0.0602 (9)	0.0517 (9)	−0.0082 (7)	0.0057 (7)	0.0040 (7)
C21	0.0678 (12)	0.0881 (13)	0.0785 (13)	−0.0158 (10)	−0.0143 (10)	0.0097 (10)

### Geometric parameters (Å, °)

**Table:**

O1—C2	1.371 (2)	C9—C13	1.370 (2)
O1—C1	1.413 (2)	C9—C10	1.475 (2)
O2—C10	1.199 (2)	C11—C12	1.479 (3)
O3—C10	1.327 (2)	C11—H11A	0.9700
O3—C11	1.457 (2)	C11—H11B	0.9700
N1—N2	1.3442 (17)	C12—H12A	0.9600
N1—C9	1.358 (2)	C12—H12B	0.9600
N1—C8	1.464 (2)	C12—H12C	0.9600
N2—C14	1.341 (2)	C13—C14	1.396 (2)
C1—H1A	0.9600	C13—H13	0.9300
C1—H1B	0.9600	C14—C15	1.471 (2)
C1—H1C	0.9600	C15—C16	1.379 (2)
C2—C7	1.375 (2)	C15—C20	1.387 (2)
C2—C3	1.383 (2)	C16—C17	1.381 (2)
C3—C4	1.372 (2)	C16—H16	0.9300
C3—H3	0.9300	C17—C18	1.382 (2)
C4—C5	1.388 (2)	C17—H17	0.9300
C4—H4	0.9300	C18—C19	1.374 (2)
C5—C6	1.379 (2)	C18—C21	1.509 (2)
C5—C8	1.505 (2)	C19—C20	1.377 (2)
C6—C7	1.383 (2)	C19—H19	0.9300
C6—H6	0.9300	C20—H20	0.9300
C7—H7	0.9300	C21—H21A	0.9600
C8—H8A	0.9700	C21—H21B	0.9600
C8—H8B	0.9700	C21—H21C	0.9600
			
C2—O1—C1	118.00 (15)	O3—C11—C12	106.82 (18)
C10—O3—C11	115.99 (16)	O3—C11—H11A	110.4
N2—N1—C9	111.65 (13)	C12—C11—H11A	110.4
N2—N1—C8	117.54 (14)	O3—C11—H11B	110.4
C9—N1—C8	130.63 (14)	C12—C11—H11B	110.4
C14—N2—N1	105.56 (12)	H11A—C11—H11B	108.6
O1—C1—H1A	109.5	C11—C12—H12A	109.5
O1—C1—H1B	109.5	C11—C12—H12B	109.5
H1A—C1—H1B	109.5	H12A—C12—H12B	109.5
O1—C1—H1C	109.5	C11—C12—H12C	109.5
H1A—C1—H1C	109.5	H12A—C12—H12C	109.5
H1B—C1—H1C	109.5	H12B—C12—H12C	109.5
O1—C2—C7	124.91 (15)	C9—C13—C14	105.70 (14)
O1—C2—C3	115.53 (15)	C9—C13—H13	127.1
C7—C2—C3	119.57 (15)	C14—C13—H13	127.1
C4—C3—C2	120.29 (16)	N2—C14—C13	110.41 (13)
C4—C3—H3	119.9	N2—C14—C15	119.64 (13)
C2—C3—H3	119.9	C13—C14—C15	129.95 (14)
C3—C4—C5	121.11 (14)	C16—C15—C20	117.46 (14)
C3—C4—H4	119.4	C16—C15—C14	121.64 (14)
C5—C4—H4	119.4	C20—C15—C14	120.90 (14)
C6—C5—C4	117.76 (15)	C15—C16—C17	120.77 (16)
C6—C5—C8	121.24 (15)	C15—C16—H16	119.6
C4—C5—C8	120.97 (14)	C17—C16—H16	119.6
C5—C6—C7	121.74 (16)	C16—C17—C18	121.67 (16)
C5—C6—H6	119.1	C16—C17—H17	119.2
C7—C6—H6	119.1	C18—C17—H17	119.2
C2—C7—C6	119.52 (15)	C19—C18—C17	117.44 (15)
C2—C7—H7	120.2	C19—C18—C21	120.94 (16)
C6—C7—H7	120.2	C17—C18—C21	121.62 (16)
N1—C8—C5	112.58 (12)	C18—C19—C20	121.25 (15)
N1—C8—H8A	109.1	C18—C19—H19	119.4
C5—C8—H8A	109.1	C20—C19—H19	119.4
N1—C8—H8B	109.1	C19—C20—C15	121.40 (15)
C5—C8—H8B	109.1	C19—C20—H20	119.3
H8A—C8—H8B	107.8	C15—C20—H20	119.3
N1—C9—C13	106.68 (13)	C18—C21—H21A	109.5
N1—C9—C10	123.21 (15)	C18—C21—H21B	109.5
C13—C9—C10	130.11 (16)	H21A—C21—H21B	109.5
O2—C10—O3	124.36 (17)	C18—C21—H21C	109.5
O2—C10—C9	125.51 (19)	H21A—C21—H21C	109.5
O3—C10—C9	110.13 (15)	H21B—C21—H21C	109.5
			
C9—N1—N2—C14	0.47 (16)	C13—C9—C10—O2	−179.11 (18)
C8—N1—N2—C14	176.15 (12)	N1—C9—C10—O3	−179.23 (14)
C1—O1—C2—C7	5.1 (3)	C13—C9—C10—O3	1.0 (2)
C1—O1—C2—C3	−174.62 (18)	C10—O3—C11—C12	172.00 (18)
O1—C2—C3—C4	−179.36 (15)	N1—C9—C13—C14	0.54 (17)
C7—C2—C3—C4	0.9 (2)	C10—C9—C13—C14	−179.66 (16)
C2—C3—C4—C5	0.1 (2)	N1—N2—C14—C13	−0.10 (16)
C3—C4—C5—C6	−1.3 (2)	N1—N2—C14—C15	−179.65 (12)
C3—C4—C5—C8	176.70 (14)	C9—C13—C14—N2	−0.28 (17)
C4—C5—C6—C7	1.5 (2)	C9—C13—C14—C15	179.21 (14)
C8—C5—C6—C7	−176.49 (15)	N2—C14—C15—C16	−167.43 (15)
O1—C2—C7—C6	179.58 (16)	C13—C14—C15—C16	13.1 (2)
C3—C2—C7—C6	−0.7 (3)	N2—C14—C15—C20	12.6 (2)
C5—C6—C7—C2	−0.5 (3)	C13—C14—C15—C20	−166.84 (15)
N2—N1—C8—C5	−86.72 (17)	C20—C15—C16—C17	0.5 (3)
C9—N1—C8—C5	88.0 (2)	C14—C15—C16—C17	−179.41 (16)
C6—C5—C8—N1	−116.20 (17)	C15—C16—C17—C18	0.5 (3)
C4—C5—C8—N1	65.9 (2)	C16—C17—C18—C19	−1.4 (3)
N2—N1—C9—C13	−0.65 (17)	C16—C17—C18—C21	177.98 (18)
C8—N1—C9—C13	−175.60 (15)	C17—C18—C19—C20	1.3 (3)
N2—N1—C9—C10	179.54 (14)	C21—C18—C19—C20	−178.05 (17)
C8—N1—C9—C10	4.6 (2)	C18—C19—C20—C15	−0.3 (3)
C11—O3—C10—O2	3.2 (3)	C16—C15—C20—C19	−0.6 (2)
C11—O3—C10—C9	−176.93 (14)	C14—C15—C20—C19	179.35 (14)
N1—C9—C10—O2	0.7 (3)		
